# A clinical method for estimating the modulus of elasticity of the human cornea *in vivo*

**DOI:** 10.1371/journal.pone.0224824

**Published:** 2020-01-08

**Authors:** David C. Pye

**Affiliations:** School of Optometry and Vision Science, University of New South Wales, Sydney, Australia; Bascom Palmer Eye Institute, UNITED STATES

## Abstract

**Background:**

To develop a method, using current clinical instrumentation, to estimate the Young’s modulus of the human cornea in vivo.

**Methods:**

Central corneal curvature (CCC), central corneal thickness(CCT), intraocular pressure (IOP) was measured with the Goldmann tonometer (IOPG) and the Pascal Dynamic Corneal Tonometer(PDCT) in one eye of 100 normal young human subjects (21.07 ± 2.94 years) in vivo. The Orssengo and Pye algorithm was used to calculate the Young’s modulus of the corneas of these subjects.

**Results:**

The Young’s modulus(E) of the corneas of the subjects using the PDCT and IOPG results (Ecalc) was 0.25 ± 0.10MPa, and without the PDCT results (Eiopg) was 0.29 ± 0.06MPa. The difference in these results is due to the difference in tonometry results between the two instruments, as the mean PDCT result for the subjects was 16.89 ± 2.49mmHg and the IOPG result 15.06 ± 2.71mmHg. E was affected by the CCC of the subjects but more particularly by the CCT and IOP measurements. Corneal stiffness results are also presented.

**Conclusion:**

Two methods have been developed to estimate the Young’s modulus of the human cornea in vivo using current clinical instrumentation. One method (Ecalc) is applicable to the general corneal condition, and Eiopg to the normal cornea, and these results can be used to calculate corneal stiffness.

## Introduction

There has been an increasing interest in the biomechanical behaviour of the eye[1], and especially of the cornea as demonstrated by Dupps and Roberts[2]. The biomechanical behaviour of the cornea may be particularly relevant in clinical practice, as the cornea may be able to be considered as a surrogate for what might be happening elsewhere in the eye[3]. Corneal biomechanics have been reported to be altered in myopic patients, and with the degree of myopia[4], be combined with baseline age to predict the rate of axial elongation in myopic children[5], be altered in diabetic patients[6], in patients using topical prostaglandins[7] and patients with keratoconus[8] and as a sensitive marker of the ocular activity of collagen vascular diseases[9]. As a result, the study of corneal biomechanics has emerged as a very hot topic for research in ophthalmology[10].

One of the key values for the biomechanical behaviour of a tissue is a measurement of the way the tissue behaves when subjected to an applied load (or stress). This gives rise to a stress/strain ratio called Young’s modulus, and this is a key value to be incorporated into engineering models of the cornea which include corneal topography, intraocular pressure and other characteristics of the eye to enable the behaviour of the tissue to be better understood.

There have been a number of methods used to try to establish the biomechanical behaviour of the human cornea ex vivo including strip extensiometry[11] and inflation testing[12,13,14]. More recent ex vivo techniques include supersonic shear imaging[15]radial shear speckle pattern interferometry[16], atomic force microscopy[17], corneal optical coherence elastography[18]and a Surface Force Apparatus[19].

There are two clinical instruments currently available to investigate the biomechanical behaviour of the cornea, the Ocular Response Analyzer and the Corvis ST. Both devices employ a puff of air of short duration to investigate corneal behaviour, but these instruments do not currently produce a measurement of Young’s modulus of the tissue.

There are, however, techniques under development which may enable a value for corneal tissue elasticity to be obtained for the human cornea in vivo. These instruments include Brilluoin optical microscopy[20,21], corneal indentation[22], estimation of Young’s modulus based on a fluid-filled spherical shell model with Scheimpflug imaging[23] and ultrasound surface wave elastography[24].

However, many of these techniques are still experimental and not available in the clinical environment. There is a need to develop a method to estimate the modulus of elasticity of the cornea using currently available clinical instruments which most ophthalmic practitioners would already have available to them in their practices. Having this knowledge may enable practitioners to better understand corneal behaviour, and how the cornea may be affected by corneal surgical techniques or rigid contact lenses. But the application of this information could be helpful for tissue matching for corneal grafts, modelling of new surgical techniques, interpreting tonometry results and determining whether the cornea may be a surrogate for what may be happening elsewhere in the eye.

In this study it is investigated whether current standard clinical instrumentation may be used to estimate the Young’s modulus of the human cornea in vivo using two methods: one to provide an accurate estimation in all corneas, and the other to provide an estimation in normal corneas.

## Materials and methods

### Subjects

One hundred subjects (100 eyes) aged between 17 and 30 years were recruited form the student and staff population of the University of New South Wales School of Optometry and Vision Science over a 3-month period, selected without a preference for gender or race. The subjects in this experiment were not the same subjects as used in the Hamilton and Pye (2008)[25] paper, and a different group of researchers collected the data. Subjects were examined and excluded from the study if they had ocular or systemic disease, a history of ocular trauma or surgery, were using ocular medications or had corneal astigmatism >2.00D. Contact lens wearers of soft contact lenses were accepted into the study provided they had not worn lenses on the day of participation in the study.

The study was conducted in accordance with the declaration of Helsinki and approved by the Human Research Ethics Committee at the University of New South Wales. Written informed consent was obtained from all subjects after a full explanation of the nature of the study.

### Procedure

Central corneal curvature (two readings, EyeChek autokeratometer, Reichert Ophthalmic Instruments, NY), applanation IOP (three readings, slit-lamp based Goldmann tonometer), Pascal DCT IOP (Swiss Microtechnology AG, Ziemer Ophthalmic Systems Group Co, Port Switzerland, three readings of Q3 or above) and ultrasonography CCT (three readings, BV International, Clermont-Ferrand, France) were measured in both eyes of each subject, with a maximum interval of 5 minutes between the use of each instrument. No measurements were taken within the first 2 hours of the subjects awakening to allow for overnight corneal swelling to dissipate, and the mean values for each instrument were used for analysis. Each procedure was conducted by a dedicated experienced independent observer who was masked from the measurements obtained from the other procedures. All results obtained for both eyes were statistically significantly correlated with p<0.0001 using the paired, two tailed t-test. As a result, only the results for one eye (the left eye) are discussed in this paper in order to avoid statistical bias from the use of both eyes of the subjects.

The baseline demographics are summarized in Table 1.

**Table 1:** Baseline demographics.

**Parameter**	**Mean (± SD)**
No. of subjects	100
Male:Female	42:58
Age (years)	21.07 (±2.94)
Central corneal curvature (mm)	7.75 (± 0.26)
Central corneal thickness (μm)	549.9 (± 32.8)
Pascal DCT IOP (mmHg)	16.89 (± 2.49)
Goldmann IOP (mmHg)	15.06 (± 2.71)

### Calculations

The Orssengo-Pye algorithm[25,26] was used to calculate the elastic (or Young’s) modulus of the cornea. These equations have been discussed in detail elsewhere, with the key equations being:

1. The Pascal DCT tonometer(PDCT) is a tonometer whose intraocular pressure results in the human eye appear to not be influenced by the material properties of the cornea of the eye being measured[27,28,29].This is due to the design of the probe tip, and the Pascal DCT tonometry result is thought to be a better indication of the true intraocular pressure, and hence the PDCT result is used in the equations below as the true IOP.
2. B is the coefficient of the Goldmann tonometry result (IOPG) of the calibration cornea (no units) and C is the coefficient of the Pascal DCT tonometry result (IOPT) in the test cornea (no units). R = average radius of curvature of the anterior cornea (mm); t = central corneal thickness (mm); ν = Poisson’s ratio of the cornea = 0.49 (no units) and A = area of applanation of the cornea (mm^2^).
  $$B=\frac{0.6\pi R(R-\frac{t}{2})\sqrt{1-\nu^{2}}}{t^{2}}$$
  $$C=\frac{\pi R{\left(R-\frac{t}{2}\right)}^{2}(1-\nu )}{At}$$
3. IOPTcalc was calculated from the equation
  $$IOPTcalc=\frac{IOPG}{(B_{c}-C_{c}+C)/B}$$
  Where IOPTcalc is the calculated true IOP (mmHg): IOPG is the Goldmann tonometer intraocular pressure (mmHg); B_c_ is the coefficient of IOPG of the calibration cornea (no units); C_c_ is the coefficient of the calibration cornea (no units); B is the coefficient of IOPG in the test cornea (no units); and C is the coefficient of IOPT in the test cornea (no units).
4. Young’s modulus (Ecalc, MPa) was calculated in vivo using the Pascal DCT tonometry value (IOPT, mmHg) in the following equation.
  $$Ecalc=\frac{\left(B.IOPG-C.IOPT\right)}{7500}$$
5. Eiopg was calculated using the above equation but using the Orssengo and Pye calculated IOPT value (IOPTcalc), and hence Step 4 enabled Ecalc and Eiopg to be calculated for the 100 subjects.
6. Step 3 enabled Ecalc and Eiopg to be plotted against IOPG and IOPT separately.
7. As the values for Ecalc and IOPG were normally distributed, a linear regression and Pearson correlation coefficient was determined (r = 0.749, p<0.0001). The linear regression equation was used to then determine the error in IOPG at differing CCTs.
8. The Orssengo and Pye algorithm was used to determine the predicted effect of CCT of the subjects on the Eiopg results.

### Statistics

GraphPad Prism (Graph Pad) version 7.02 and Excel (Excel for Office 365) was used to perform the statistical analysis. All tests were parametric and two-tailed, with statistical significance set at p<0.05.

## Results

### Distribution of Young’s modulus

**Fig 1:**
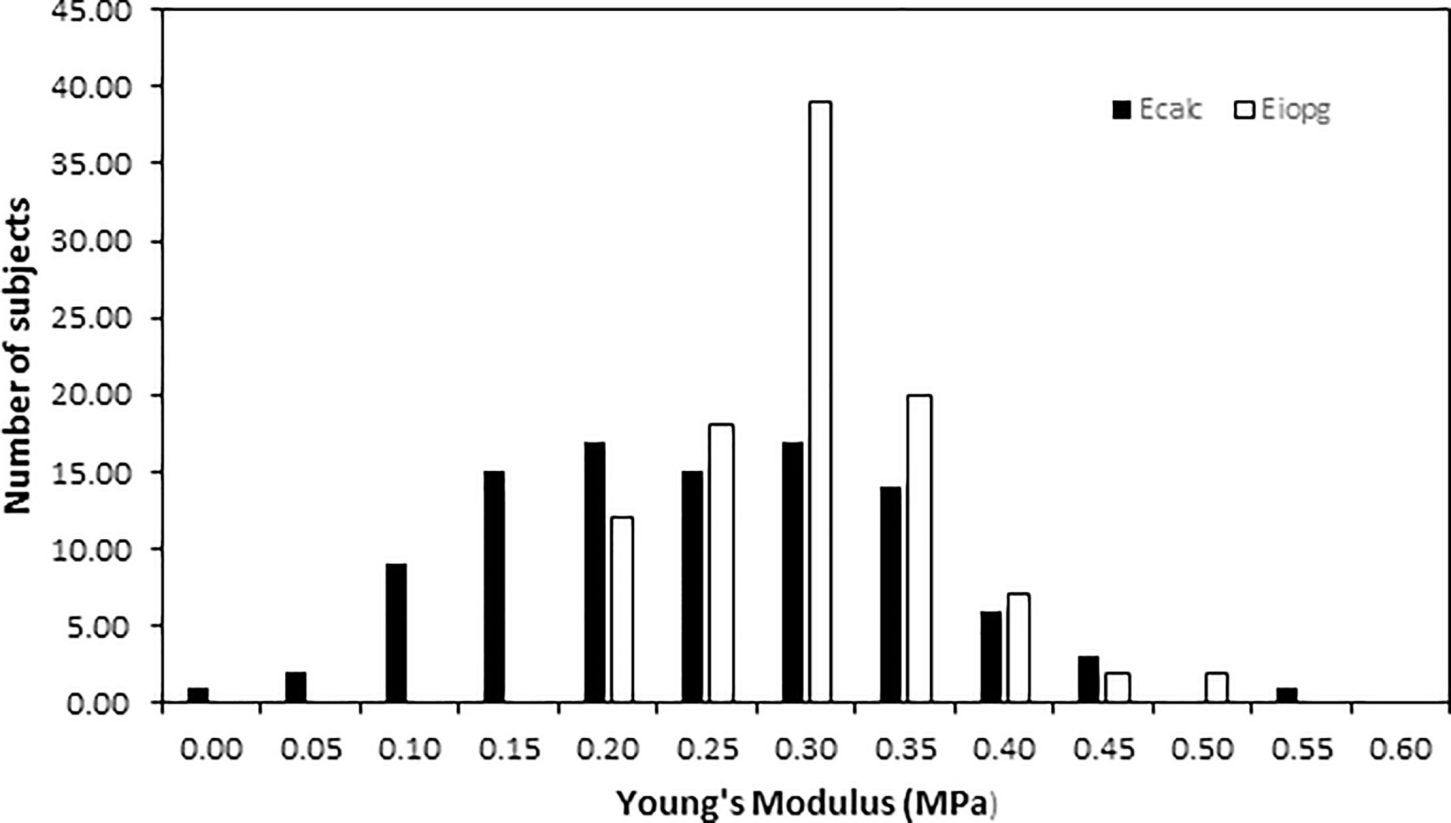
Distribution of Young’s modulus in 100 young healthy eyes as calculated using the Orssengo and Pye formula and Goldmann tonometry results only (Eiopg) or Goldmann and Pascal DCT results combined (Ecalc).

The mean value and standard deviation of Ecalc for the subject group was 0.25 ± 0.10 MPa and for Eiopg was 0.29 ± 0.06 MPa. The data for both values is normally distributed and the frequency distribution is shown in Fig 1.

### Intercorrelation of parameters

A summary of the intercorrelations of parameters is shown in Table 2.

**Table 2:** Significance of Pearson correlations for the variables (* = p < 0.05, ** = p < 0.001).

	CCC (mm)	IOPG (mmHg)	PDCT (mmHg)	IOPTcalc (mmHg)	CCT (mm)	Ecalc (MPa)	Eiopg (MPa)
**CCC (mm)**	----------	ns	ns	ns	*	*	*
**IOPG (mmHg)**	ns	----------	**	**	ns	**	**
**PDCT (mmHg)**	ns	**	----------	**	ns	ns	**
**IOPTcalc** **(mmHg)**	ns	**	**	----------	**	**	**
**CCT** **(mm)**	*	ns	ns	**	----------	**	**
**Ecalc** **(MPa)**	*	**	ns	**	**	----------	**
**Eiopg** **(MPa)**	*	**	**	**	**	**	----------

CCC was negatively correlated with Ecalc (r = -0.199, p = 0.0467) and Eiopg (r = -0.197, p = 0.0492) but not IOPG, PDCT or IOPTcalc.

**Fig 2:**
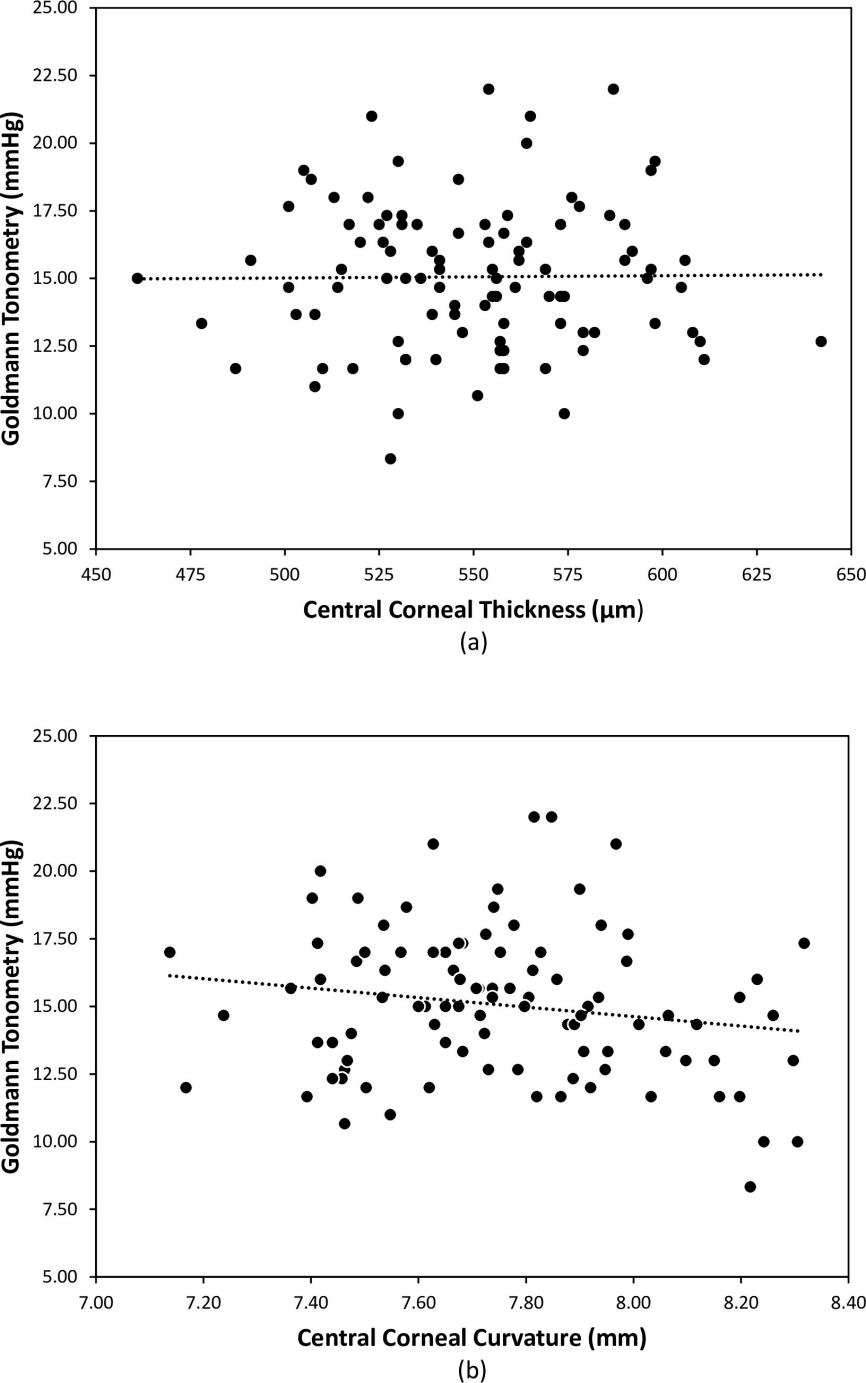
Correlation between Goldmann IOP and (a) central corneal thickness (r = 0.010, p = 0.921); (b) central corneal curvature (r = -0.169, p = 0.092).

There was no significant Pearson correlation between IOPG and CCT (r = 0.010, p = 0.921) as shown in Fig 2A, nor IOPG and central corneal curvature (CCC) (r = -0.169, p = 0.093) as shown in Fig 2B. IOPG was significantly correlated with PDCT (r = 0.5969, p<0.0001), Ecalc (r = 0.7468, p<0.0001), Eiopg (r = 0.900, p<0.0001) and IOPTcalc (r = 0.900, p<0.0001).

The PDCT results were significantly correlated with Eiopg (r = 0.526, p<0.0001) and IOPTcalc (r = 0.526, p<0.0001) but not with CCT, CCC or Ecalc results, suggesting that the PDCT IOP is importantly independent of these three variables.

**Fig 3:**
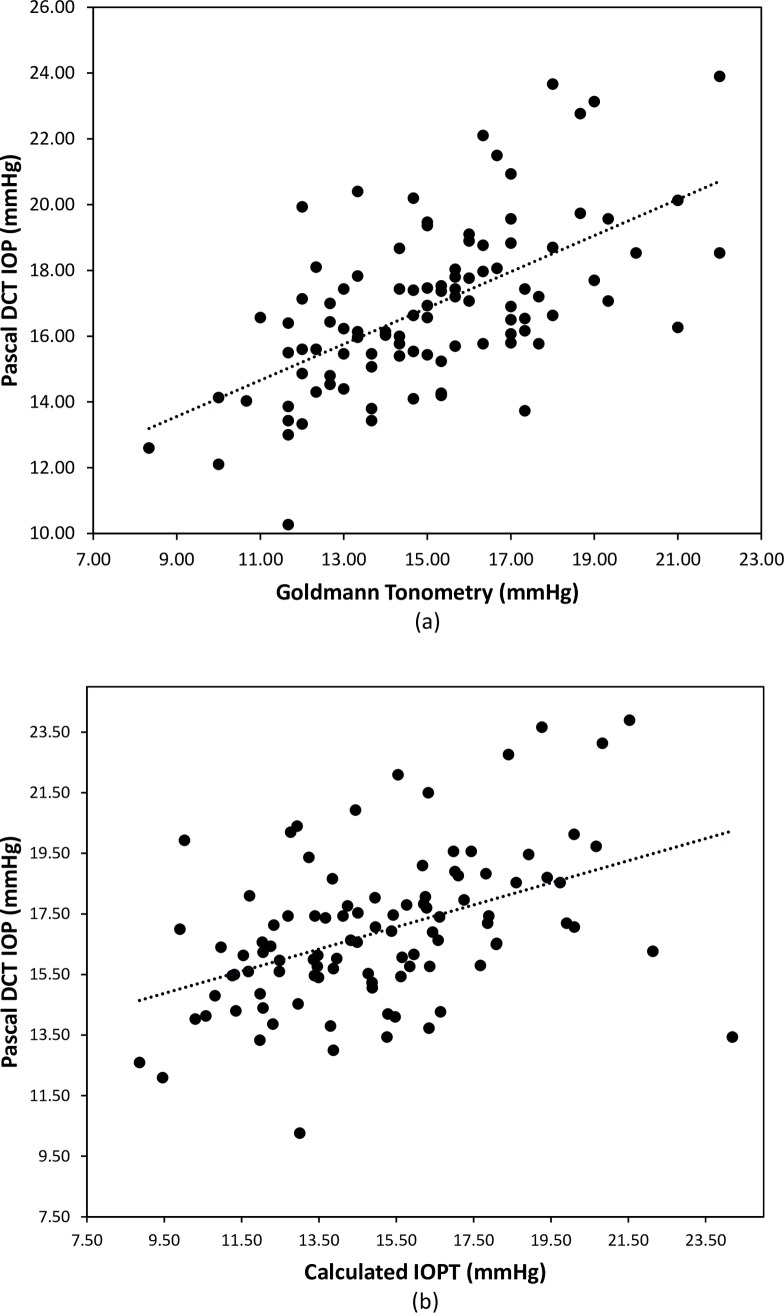
Correlation between Pascal DCT and (a) Goldmann IOP (r = 0.597, p < 0.001); (b) calculated IOPT (r = 0.525, p < 0.001).

There was a positive correlation between IOPG and PDCT (r = 0.598, p<0.001, Fig 3A), PDCT and IOPTcalc (r = 0.525, p<0.001, Fig 3B), with the mean PDCT result higher than the IOPTcalc by 1.99mmHg.

IOPTcalc was positively correlated with IOPG (r = 0.900, p<0.0001), and negatively correlated with PDCT (r = 0.526, p<0.0001) and CCT (r = -0.413, p<0.001), but not correlated with CCC.

CCT was positively correlated with CCC (r = 0.230, p<0.05), negatively correlated with IOPTcalc (r = -0.4131, p<0.0001), Ecalc (r = -0.502, p<0.0001) and Eiopg (r = -0.409, p<0.0001), but not correlated with IOPG or PDCT.

**Fig 4:**
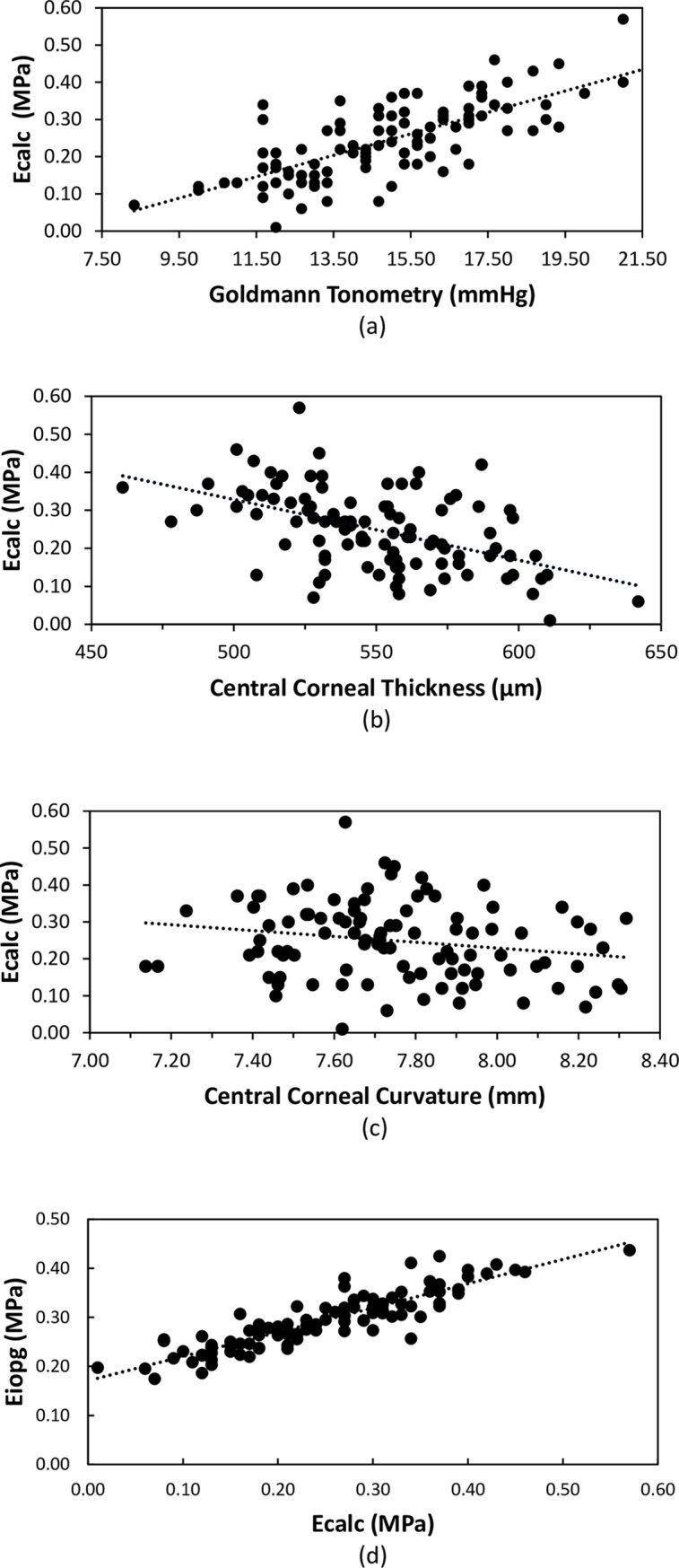
Correlation between Ecalc and (a) Goldmann IOP (r = 0.748, p < 0.001); (b) central corneal thickness (r = -0.502, p < 0.001); (c) central corneal curvature (r = -0.199, p = 0.047); (d) Eiopg (r = 0.890, p < 0.001).

**Fig 5:**
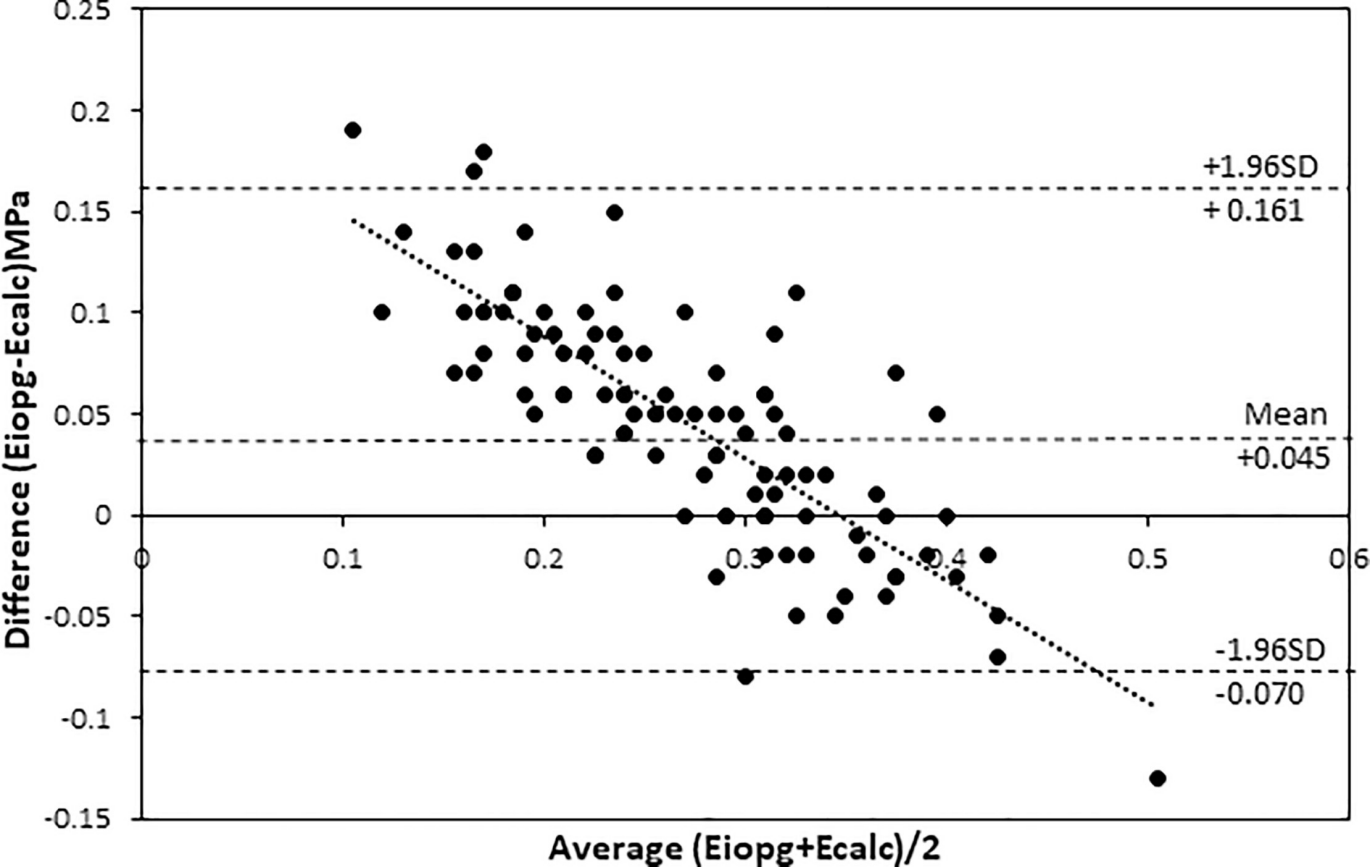
Bland Altman plot of the relationship between Ecalc and Eiopg demonstrating proportional bias.

Young’s modulus (Ecalc) was positively correlated with IOPG (r = 0.749, p<0.001, Fig 4A). Ecalc was also correlated to CCT (r = -0.502, p<0.001, Fig 4B) and weakly to corneal curvature (r = -0.199, p = 0.05, Fig 4C). The latter correlation should be viewed with suspicion, as this relationship was not found in the contralateral eye (r = -0.11, p = 0.27). Ecalc was correlated with Eiopg (r = 0.890, p<0.001, Fig 4D), but there is proportional bias as shown in the Bland Altman plot[27]in Fig 5. However, Ecalc was not correlated with PDCT (r = 0.093, p = 0.36).

Eiopg was positively correlated with PDCT (r = 0.526, p<0.001), IOPTcalc (r = 0.998, p<0.001), Ecalc (0.890, p<0.0001) and IOPG (r = 0.900, p<0.001) and negatively correlated with CCC (r = -0.197, p = 0.049) and CCT (r = -0.409, p<0.0001).

## Discussion

### 1. CCT vs CCC

The association between CCT and CCC was not strong, but was statistically significant in both eyes of the subjects. The finding that thicker corneas tended to have flatter CCCs has also been reported by Kiely et al[28], Hovding[29], and Tomidokoro[30]. Studies reported by Lowe[31], Tomlinson[32] and Hamilton and Pye[25] found no correlation between CCCT and CCC, and their study was conducted with similar instrumentation and similar population to that reported in this study.

### 2. Goldmann vs Pascal DCT tonometry

The tonometer recognised by the International Standards Organisation[33] as the reference tonometer is the Goldmann tonometer, and yet in 1993 Whitacre and Stein[34] identified as many as 26 potential sources of error with this tonometry technique. The PDCT tonometer claims to be independent of corneal properties due to the design of the footplate and contour matching of the corneal front surface, and the results with this tonometer have been shown to be in good agreement with manometer studies conducted ex vivo and in vivo[35,36,37].

In 19 of the subjects, the difference in IOP measured by the Goldmann and PDCT was ≥4mmHg and, in most instances, PDCT IOP was greater than IOPG, and this difference contributed to the larger spread of results for Ecalc as shown in Fig 1.

If the Goldman and PDCT tonometers are accurate measures of a patient’s true IOP, it would be expected that their tonometry results would be equivalent for the mean CCT and CCC ie the average eye values for this particular subject group. However, the PDCT values were 1.8mmHg higher than the those of IOPG. This discrepancy has been noted in the past, and previous studies have found PDCT values to be higher than IOPG by 0.8mmHg[38], 1mmHg[39], 1.7mmHg[40], 2.3mmHg[41], 2.34mm Hg[42] and 3.98mmHg[43]. This difference in results between the two instruments has been found to be linked to the corneal resistance factor measured by the Ocular Response Analyzer by Kotecha et al[41], but another possible cause for this IOP measurement difference might be the surface tension developed at the edge of the Goldmann prism when measuring IOP, which should not apply to the PDCT technique. There are published works which suggest that the surface tension of the tear film for the GAT probe might vary from 0.11mmHg[44,45], 0.26mmHg[46] 4.15mmHg[47], 4.67mmHg[48], to 4.7mmHg[49] and hence a difference of 1.9mmHg between the two tonometry techniques could be as a result of surface tension acting during the IOPG measurement.

Hamilton and Pye[38] (2008) felt that the difference in mean values between the Pascal DCT and Goldmann tonometers could possibly be explained by slight calibration differences between the two instruments, interobserver variations or population differences. The difference between the IOP measurements determined by Goldmann and PDCT were not correlated with CCC (r = -0.07, p = 0.55).

### 3. Young’s modulus of the cornea in vivo:

In this paper, the calculation of Young’s modulus is based upon the cornea acting as a linear elastic material on the basis of a small applied load (around 1.5gf) and a small corneal indentation by the Goldmann tonometry probe of approximately 150μm.

There are two values for Young’s modulus presented in this paper. Ecalc is based on the clinical measurements of a patent’s CCC, CCT, IOPG and PDCT, and it is assumed that the PDCT result is a true measure of IOP and that IOPG is a measure of the cornea’s response to a specific applied load.

Eiopg was calculated from the CCC, CCT and IOPG results, and is based on the assumption that IOPG is equivalent to IOPT when the cornea has average values for CCT and CCC (called the calibration cornea (Orssengo and Pye)[26]. As CCC has little effect on IOPG results, Eiopg can be calculated in clinical practice from the CCT and IOPG results alone.

The results for Eiopg and Ecalc are similar to those reported in a similar cohort of subjects reported in other papers and these results are shown in Table 3 for comparison.

**Table 3:** Published values for the Young’s modulus for human corneas of human subjects of similar age to those in this study.

Author	Technique	In vivo or ex vivo	Number of subjects	Age of subjects (years)	Young’s modulus (MPa) (±SD)
**Hamilton and Pye 2008^25^**	Clinical measurements and Orssengo-Pye algorithm	In vivo	100	22.0±2.9	Eiopg 0.29± 0.06
**Knox Cartwright et al 2011^16^**	Radial shearing speckle pattern interferometer	Ex vivo	1	24	0.28
**Lam et al 2015^22^**	Corneal indentation	In vivo	29	23.4±1.7	Etangent 0.76±0.16
**Shih et al 2015^23^**	Corvis ST based on water-filled spherical diaphragm dynamics	In vivo	10	25.2±2.15	0.44±0.37
**Sit et al 2017^24^**	Ultrasound surface wave elastography	In vivo	20	51.4±7.2	0.69± 0.11
**Pye 2019**	Clinical measurements Orssengo-Pye algorithm	In vivo	100	21.1±2.0	Ecalc 0.25±0.10 Eiopg 0.29±0.06

Ecalc and Eiopg are strongly correlated (r = 0.890, p<0.001) but Ecalc was, on average, 0.04MPa less than Eiopg. Hence the results are not interchangeable but, in the clinical situation, Eiopg may be a useful indicator of Young’s modulus when determined with healthy normal corneas. However, if the IOPG result is likely to be significantly affected by the physiological state of the cornea such as oedema[50,51], refractive surgery[52,53] or other causes, Ecalc should provide a more valid result.

**Fig 6:**
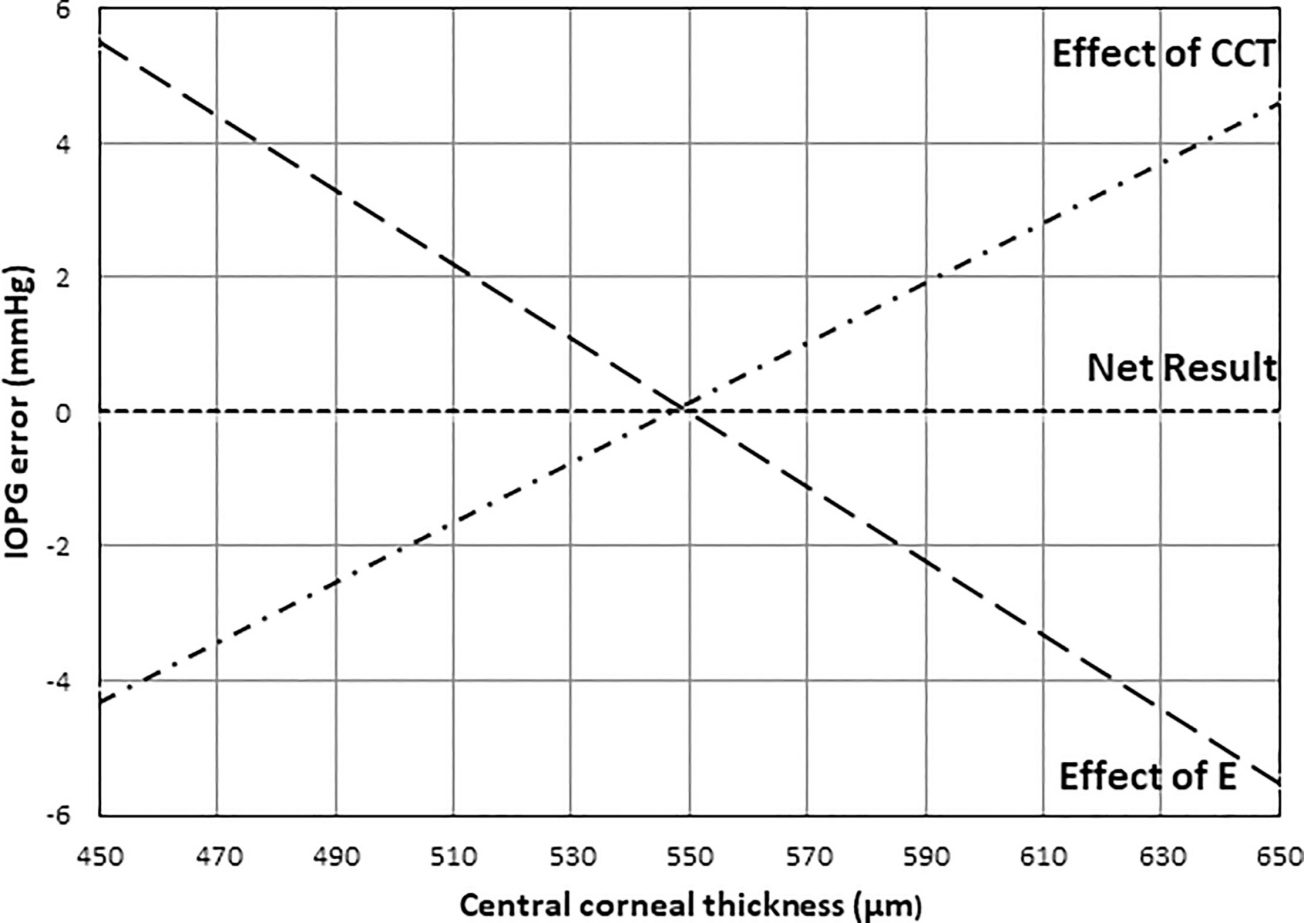
Figure demonstrates the actual Goldmann tonometry error obtained in this subject group (Net Result), the Goldmann tonometry error calculated from E iopg (Effect of E) and from the error due to central corneal thickness as calculated from the Orssengo and Pye (Effect of CCT).

In the young healthy adults reported in this study, it was surprising that there was no apparent significant effect of CCT on IOPG as shown in Fig 2, but Ecalc had a significant effect upon IOPG as shown in Fig 4A, with Ecalc decreasing as CCT increases. By using the data from this study and the Orssengo and Pye algorithms[26], the effects of Ecalc and CCT upon IOPG can be calculated, and are shown in Fig 6. Over the 200μm range of corneal thicknesses presented in Fig 6, the total effect of central corneal thickness on IOPG was estimated as 8.9mmHg, and of Ecalc was 11.0mmHg. It is apparent that Ecalc has a greater effect on IOPG measurement error than CCT, and this confirms a similar finding published by Hamilton and Pye[20]. In the young healthy normal subject group of this study, the measurement error in IOPG predicted by changes in CCT are negated by changes in Ecalc, supporting the predictions regarding the significant effect of Young’s modulus on IOPG made by Liu and Roberts[54].

The above effect of Ecalc on IOPG could be related to the behaviour of the number of layers of corneal collagen or the effect of hydration upon the biomechanical behaviour of the cornea and the subsequent effects on IOPG as discussed by Hatami-Marbini and Etebu[55].

**Fig 7:**
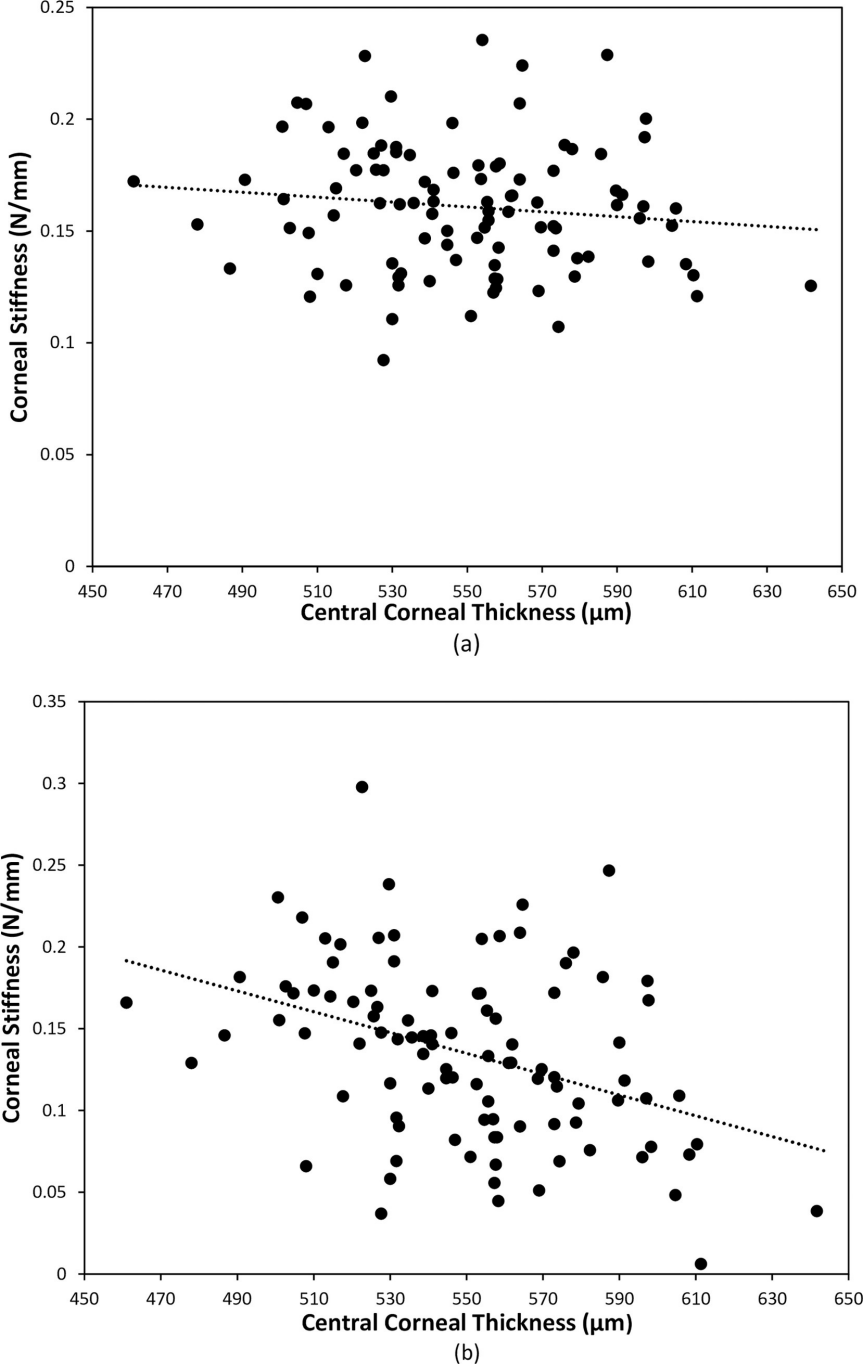
Correlation between corneal stiffness as calculated from Eiopg and (a) central corneal thickness (r = -0.123, p = 0.224); (b) Ecalc and central corneal thickness (r = -0.388, p < 0.001).

However, these results appear to be related the structural stiffness of the cornea, which has been described by Palko and Liu[56] by the equation k = t.E, where k = stiffness, t = the central corneal thickness and E is the Young’s modulus of the tissue. If k is calculated using Eiopg and CCT, there is no correlation between CCT and k as shown in Fig 7A, and k = 0.16 ± 0.03 N/mm. If k is calculated using Ecalc, there is significant relationship between the two variables (p<0.001), but there is considerable scatter of the data as shown in Fig 7B.

The corneal stiffness results suggest that applanation tonometry results in young healthy normals are influenced by the corneal stiffness ie the CCT and E combined rather than one variable alone. Whether this relationship applies in a similar fashion for subjects in older age groups is yet to be investigated.

The value for k of 0.16N/mm is greater than the value of 0.079N/mm obtained by Lam et al[22] who used a corneal indentation device. Whilst Lam et al[22] used a similarly aged subject cohort, their sample size was considerably smaller than in this study, and the cornea was indented to depth of 1mm, which is greater than that which occurs during Goldmann tonometry. It might also be possible that the anterior cornea is stiffer than the cornea as a whole, and this could also contribute to the discrepancy in values.

**Fig 8:**
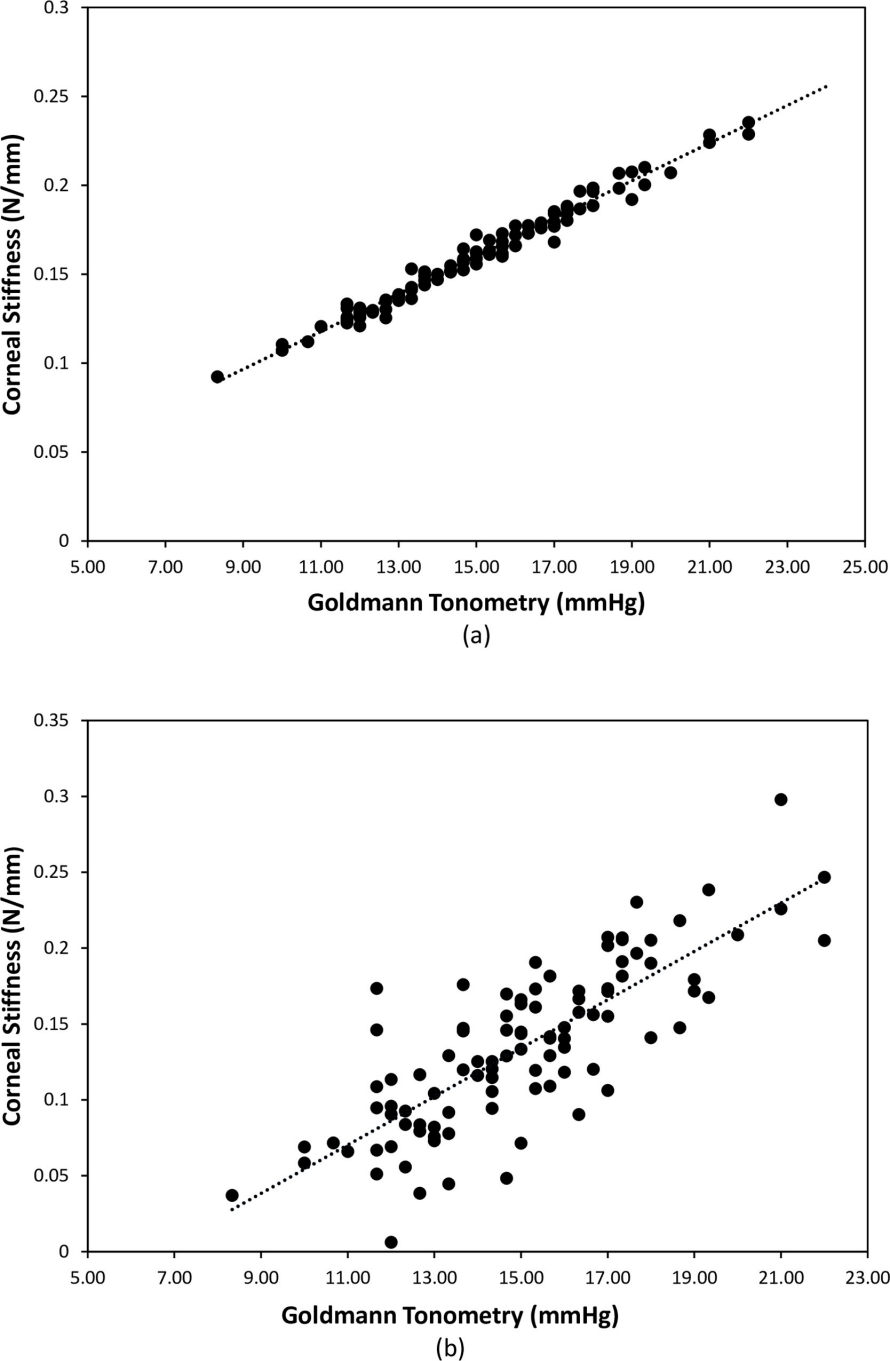
Correlation between corneal stiffness and IOPG calculated from (a) Eiopg (r = 0.989, p < 0.001); (b) Ecalc (r = 0.801, p< 0.001).

As can be seen from Fig 8A, there is a very strong relationship between corneal stiffness (calculated from Eiopg and CCT) and the Goldmann tonometry values. This strong correlation may be expected as the IOPG results were used in the calculation of these corneal stiffness values, but this result indicates the strength of the relationship between corneal stiffness and IOPG. There is a similarly strong correlation between IOPG and corneal stiffness calculated using Ecalc (r = 0.801, p < 0.001 see Fig 8B).

## Conclusion

This paper presents a linear elastic model to estimate the Young’s modulus of the human cornea in vivo, although the cornea is thought to be anisotropic, nonlinear, viscoelastic[57] and hyperelastic[58] However, many of the techniques to obtain these results above are performed ex vivo and do not use Goldmann tonometry, which applies a relatively small force (1.5 gf) over an area of 7.35mm^2^, with a small degree of central anterior corneal deformation (approximately 150μm when the measurement is taken), and the force is applied over a longer time period than the non-contact tonometers.

This paper presents two methods for estimating the Young’s modulus of the cornea in vivo which involve the use of currently available clinical instrumentation. The most accurate method is Ecalc which involves the use of the Pascal DCT as well as IOPG, and relies upon the Pascal DCT providing a more accurate measurement of the true IOP in all instances.

There is a strong correlation between Ecalc and Eiopg in young healthy adults, although the results are not interchangeable and, as CCC has no effect on Eiopg, it may be feasible to calculate Eiopg from CCT and IOPG alone in patients with normal corneas. As CCT and IOPG are often measured routinely in clinical practice, this would enable Eiopg to be calculated relatively easily in the clinical setting.

Young’s modulus is an important descriptor of the mechanical behaviour of a material and being able to estimate this value for the individual patient in clinical practice may enable further developments in corneal modelling, biomechanics and bioengineering (such as the development of artificial corneas). It may also provide further information regarding improvements to corneal surgery, comparison of tonometers and tonometry methods, methods for altering anterior surface corneal topography (surgery and contact lenses), corneal aging, early diagnosis of corneal conditions (such as keratoconus) and the effects of topical drugs on corneal behaviour and IOP measurement. The behaviour of the cornea may also be a surrogate for what is happening elsewhere in the eye, such as the management and diagnosis of glaucoma, myopia development and the effects of systemic diseases on the eye, and a number of these issues are currently being investigated.

These results suggest that corneal stiffness affects applanation tonometry results, and that both CCT and E combine to affect the findings. As such, it would be unwise to consider applying an IOPG correction factor based on only one of these two variables in subjects of this age group.

## Supporting information

S1 Dataset Data for all of the subjects in both eyes is included in this dataset. (XLSX)
